# The Clinical Significance of High Antimicrobial Resistance in Community-Acquired Urinary Tract Infections

**DOI:** 10.1155/2020/2967260

**Published:** 2020-06-04

**Authors:** Maria G. Zavala-Cerna, Midrori Segura-Cobos, Ricardo Gonzalez, Isidro G. Zavala-Trujillo, Silvia F. Navarro-Perez, Jose A. Rueda-Cruz, Fernando A. Satoscoy-Tovar

**Affiliations:** ^1^Immunology Department, International Program of Medicine, Universidad Autonoma de Guadalajra, Guadalajara, Jal, Mexico; ^2^Facultad de Biología, Instituto de Ciencias Exactas y Terrestres, Universidad Autonoma de Guadalajara, Guadalajara, Jal, Mexico; ^3^Infectious Disease Division, Hospital Angel Leaño, Universidad Autonoma de Guadalajara, Guadalajara, Jal, Mexico; ^4^Microbiology Department, Unidad de Patología Clínica, Guadalajara, Jal, Mexico

## Abstract

**Background:**

Urinary tract infections (UTIs) affect up to 150 million individuals annually worldwide, mainly due to *Escherichia coli* (*E. coli*) and *Klebsiella*. The emergence and spread of multidrug-resistant (MDR) bacteria are increasing, representing one of the biggest threats for human health. The objective of our study was to describe antimicrobial patterns of resistance and identify risk factors associated with MDR uropathogens.

**Methods:**

We conducted a cross-sectional study in 296 patients with community-acquired UTI who underwent clinical and microbiologic analysis, and clinical associations to MDR uropathogens were investigated. *Findings*. Microbiological analysis included *E. coli* (55%), ESBL-*E. coli* (26%), *Enterococcus* (6%), *Klebsiella* (5%), and others (8%). Higher frequencies of MDR bacteria were found among ESBL-*E. coli*, with resistance to ampicillin (100%), ceftriaxone (96%), gentamicin (57%), ciprofloxacin (89%), and TMP/SMX (53%). However, they were sensitive to fosfomycin (6.6%), nitrofurantoin (1.3%), and carbapenems (0%). Fosfomycin MIC90 for ESBL-*E. coli* was 5.78 *μ*g/mL. The only clinical variable with significant association to ESBL producers was the presence of comorbidities: hypertension and type 2 diabetes mellitus with an OR (95%CI) of 2.5(1.3 − 4.9)(*p* < 0.01) and 2.8(1.2 − 6.7)(*p* < 0.05), respectively.

**Conclusions:**

In the majority of cases, resistance rates to commonly prescribed antimicrobials in UTIs were high, except for fosfomycin, nitrofurantoin, and carbapenems. To provide appropriate treatment, both the identification of risk factors and the uropathogen would be important. An active surveillance in UTIs in the community is required since the proportion of ESBL producers is increasing.

## 1. Introduction

Urinary tract infections (UTIs) are defined as the presence of classical signs and/or symptoms and urine culture demonstrating the presence of known uropathogens above the given threshold (>100 cfu/ml urine to 100,000 cfu/ml urine) [1]. Although this combination is not always present, it is estimated that close to 20% of women can manifest symptoms without positive urine cultures and significant numbers of bacteria can be found in asymptomatic individuals [2, 3]. UTIs are classified as either complicated or uncomplicated; the first depends on the presence of anatomical or functional urological abnormalities, pregnancy, immunosuppression, and signs and symptoms related to tissue invasion or systemic affection [4]. UTIs affect up to 150 million individuals annually worldwide [5]; incidence varies across continents with reports of 2,400 per 100,000 women annually in Europe and over 8 million annual cases in the US. This amount of cases provided data to estimate that 1 out of 3 women will develop a UTI requiring antibiotic treatment by age of 24, and 1 out of 2 women will develop at least one episode of UTIs during their lifetime [6]. Successful bacterial invasion of the urinary tract depends on several factors, either attributable to the host, such as barrier defense mechanisms, hormone regulation of the immune system, and changes in the genital microbiota, or factors attributable to the pathogen itself such as inoculum size, virulence factors that are aimed at the adhesion or invasion of the urinary tract epithelia, and the formation of biofilms that confer protection against host immune response or antibiotic treatment [7]. Common uropathogens that cause community-associated UTIs include *Escherichia coli* (*E. coli*), *Klebsiella pneumoniae*, and *Enterococci*, with the first being the primary causative agent (80%) in most studies [8]. Furthermore, among *E. coli* isolates, it has been found that from 1.6% to 3.9% are producers of extended spectrum-beta lactamases (ESBLs) in outpatient settings reported in France and the United States, respectively [9, 10], and in our own previous experience, but with a different approach, ESBLs can be found in up to 69.5% in the outpatient setting [11]. The 2010 IDSA clinical guidelines for treatment of uncomplicated UTIs recommend selecting one of the following regimens of treatment: (1) nitrofurantoin monohydrate/macrocrystals 100 mg twice daily for 5 days; (2) TMP/SMX 160/180 mg twice daily for 3 days; (3) fosfomycin 3 mg as a single dose; (4) pivmecillinam 400 mg twice daily [12]. Uropathogens in the community setting have developed high rates of resistance, specially to quinolones carbapenems and third-generation cephalosporins, due to bacterial production of extended-spectrum-beta lactamases (ESBLs) [13], carbapenemases [14], and biofilm production, which allows the bacteria to withstand hostile environmental conditions and makes them capable of causing a broad range of chronic diseases [15]. The mechanisms for acquisition of these proteins can be either by *de novo* mutations or mobile genetic elements carrying resistance genes [16]. The emergence and spread of multidrug-resistant (MDR) bacteria is increasing worldwide, defined as resistance to at least one agent of three or more antibiotic classes, and represents one of the biggest health threats for human health, not just in the recent years, but will be a major issue in the coming decades, all the way to a devastating problem in 2050, unless we find alternatives for treatment [17]. Attributable factors for this phenomenon include limited therapeutic options, limited diagnostic facilities, inappropriate prescription practices, inadequate patient education, unauthorized sales of antimicrobials, and lack of appropriate functionating drug regulatory mechanisms [18]. While several comorbidities increase susceptibility to UTIs, most of them occur in otherwise healthy women; therefore, the objective of this work was to describe antimicrobial patterns of resistance in our community, as well as the identification of risk factors associated with the presence of multiresistant pathogens causing UTIs.

## 2. Methods

We conducted a cross-sectional study, in the occupational medicine clinic at “Unidad de Patologia Clinica” in Guadalajara, Jal., Mexico. The study was reviewed and approved by the Universidad Autonoma de Guadalajara Health Sciences Center Institutional Review Board prior to initiation.

### 2.1. Inclusion of Subjects

For the present study, we recruited subjects from the occupational clinic (provides service for several companies that have facilities in the state of Jalisco), as well as patients referred from private clinics and public health systems in Guadalajara, Jalisco.

Eligible subjects were assessed for inclusion criteria and were invited to participate in the study after explanation of the informed consent and acceptance to participate in the study.

Subjects were assessed by general practice physicians from Universidad Autonoma de Guadalajara, in the occupational clinic facilities. Inclusion criteria were subjects with suspicion of a UTIs by their treating physician, subjects >18 years old, capable of answering questionnaires, and voluntarily accepted participation in the study. Exclusion criteria were subjects that declared the intake of broad-spectrum antibiotics or being in a hospital or nursery home 3 days prior to the inclusion.

Predesigned questionnaires were used to obtain clinical data relevant for urinary tract infections. All included patients underwent urine sample collection. Cases of urinary tract infections were defined as the presence of at least one sign or symptom previously associated with the disease in combination with the presence of 10^3^ bacteria/mL or as asymptomatic bacteriuria with the sole presence of 10^5^ bacteria/mL in the urine sample without clinical manifestations. In all cases, urine samples were collected by clean catch method; samples were conserved at room temperature before they were analyzed (no more than 30 minutes). All samples underwent urinalysis and culture.

### 2.2. Microbiological Analyses

We used standard techniques for culture and pathogen identification [19]. Bacteria susceptibility testing to antimicrobial drugs were performed by microdilution methodology (VITEK 2 XL @ Biomerieux), with panels comprising different concentrations for antibiotic drugs, except for fosfomycin. Inhibition for each isolate was defined as susceptible, intermediate, or resistant, according to the guidelines of Clinical and Laboratory Standards Institute (CLSI) [20]. To identify ESBL producer strains, by phenotypic conformation, we used the AST-N286 card containing cefepime (1 mg/mL), cefotaxime (0.5 mg/mL), ceftazidime (0.5 mg/mL), cefepime/clavulanic acid (1/10 mg/mL), cefotaxime/clavulanic acid (0.5/4 mg/mL), and ceftazidime/clavulanic acid (0.5/4 mg/mL) as recommended by the CLSI. Bacterial growth at or above the screening antibiotic concentration for all penicillins and cephalosporins was confirmed as ESBL production.

The *in vitro* susceptibility to fosfomycin was measured by broth dilution and commercially available disk diffusion containing 200 *μ*g of fosfomycin and 50 *μ*g of G6P. The interpretation of fosfomycin susceptibility in *E. coli* was performed using the CLSI 2012 guidelines: susceptible (≤64 mg/mL), intermediate (128 mg/mL), or resistant (≥256 mg/mL) [20]. MIC and minimum bactericidal concentration (MBC) of fosfomycin to microbial strains was determined by broth dilution as well [21]. In brief, Mueller–Hinton agar (MHA) medium was adjusted to different concentrations of fosfomycin (5–160 *μ*g/mL) and glucose-6-phosphate (G6P) was added; then, bacterium inoculum was introduced, previously quantified (O.D600 nm = 0.1; approximately 1 × 108 per mL^−1^), and diluted 1 : 10 in saline solution. Petri dishes were incubated 18–24 hrs at 37°C. After this time, bacterial colonies were evaluated according to their growth and compared with respect to the control. Control plaques were used for bacterial growth in the absence of antibiotics to determine both bacterial growth and viability.

### 2.3. Statistical Analysis

For descriptive purposes, categorical variables are presented as numbers and percentages and continuous variables with mean ± standard deviation, unless otherwise indicated. For inference purposes, categorical variables were compared using *χ*^2^analysis, and continuous variables with normal distributions were compared using Student's *t*-test. For nonnormally distributed variables, we used Fisher's exact test. To test differences among multiple groups of bacteria for continuous variables, we used a 1-way analysis of variance (ANOVA) with Bonferroni multiple comparison test. Except for the age, the distribution of variables was non-Gaussian (Shapiro–Wilk's test, *p* > 0.1). For multiple variable analysis, we used logistic regression. The hypothesis was tested at 95% confidence interval (CI), two-sided, and the level of type I error was set at *a* = 0.05. All statistical analyses were done using STATA/IC 15.1 software.

## 3. Results

After 12 months, a total of 500 subjects were included in the study; 29 patients were excluded due to sample or clinical information loss. From 471 subjects, we were able to confirm positive urine cultures in 296 cases. From these, 247 were confirmed cases of UTIs with both clinical symptoms and the presence of 10^3^ CFU/mL, and in 49 cases, the urine culture was positive with up to ≥10^5^ CFU/mL, but there was absence of typical clinical manifestations. From these 296 confirmed cases of community-acquired UTIs and asymptomatic bacteriuria, the mean age was 54 ± 19 years; 262 (89%) were women; of these, 130 (50%) were postmenopausal and 13 (5%) were pregnant. None of the subjects reported awareness of an anatomical/functional abnormality in the urinary tract or an immunosuppressive condition.

The most common symptom reported was pollakiuria in 228 (77%), and the least common was fever in 47 (16%). We found that 83 (28%) patients referred at least one previous episode of a UTI in the current year that required the use of antibiotic treatment. During interview, we asked for the presence of comorbidities; the most frequent was hypertension in 80 (27%) of patients, followed by diabetes and hypothyroidism.  Table 1 enlists clinical variables of cases with UTIs.

After these observations, it was established that from 247 cases of UTI, 56 (23%) were complicated, either by the presence of one of the following or a combination in both pregnancy and fever.

With respect to isolated pathogens being the causative agents of community-acquired UTIs in our study, the most frequently found was *E. coli* (*n* = 240), followed by *Enterococcus faecalis* (*n* = 18), *Klebsiella pneumoniae* (*n* = 14), *Staphylococcus* sp. (*n* = 7), *Pseudomonas aeruginosa* (*n* = 6), *Proteus mirabilis* (*n* = 4), *Citrobacter freundii* (*n* = 3), *Candida albicans* (*n* = 2), *Serratia marcescens* (*n* = 1), and *Streptococcus agalactiae* (*n* = 1). From the *E. coli* isolates, 76 (25.6%) were classified as ESBL producers.

Antimicrobial resistance was tested to commonly prescribed antibiotic drugs including beta-lactams (penicillin, cephalosporins, monobactams, and carbapenems), macrolides, aminoglycosides, sulfonamides, phosphonates, nitro derivatives, and quinolones. Depending on susceptibility patterns, different numbers of antibiotics were tested for each pathogen.

**Figure 1:**
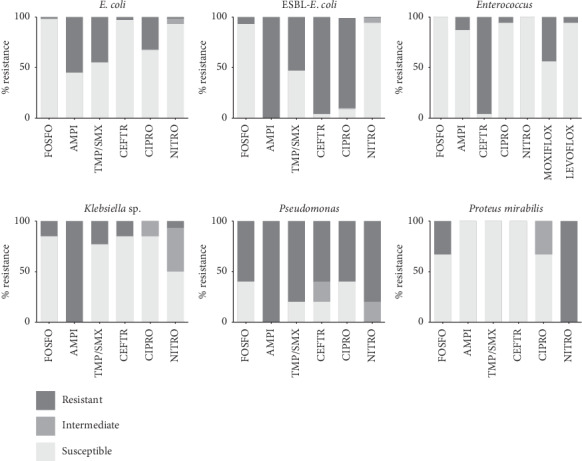
Resistance patterns in isolated uropathogens from community-associated uncomplicated cystitis to optimal treatment options according to IDSA guidelines and the European society for microbiology and infectious diseases.

Resistance rate to antimicrobials, reported in more than 20% of *E. coli* isolates, was reported to ampicillin, TMP/SMX, and ciprofloxacin. Importantly, ESBL- *E. coli* was multidrug resistant (MDR) with high resistance to ampicillin (100%), ceftriaxone (96%), gentamicin (57%), ciprofloxacin (89%), and TMP/SMX (53%). On the contrary, low resistance was identified to fosfomycin (6.6%), nitrofurantoin (1.3%), and carbapenems (0%). The highest resistance rates to nitrofurantoin were found among *Pseudomonas* and *Proteus* isolates. Frequencies of most common isolated pathogens and their antimicrobial resistances are represented in Figure 1, taking into consideration guidelines for treatment of community-associated UTIs, except for pivmecillinam, which is not yet available in Mexico. For additional and more precise information, see Supplementary Table 1.

Forty-eight strains of uropathogenic *Escherichia coli* (UPEC) and four strains of *Klebsiella pneumoniae* were randomly selected to determine the degree of susceptibility to fosfomycin. Twenty-four UPEC strains were ESBL producers, while the remaining strains were non-ESBL producers. The MIC90 of fosfomycin for the ESBL-UPEC and non-ESBL-UPEC strains was 5.78 *μ*g/ml and 5.62 *μ*g/ml, respectively. In addition, significant differences were observed in the MBC of fosfomycin for the ESBL-UPEC strains (63.75 *μ*g/ml), with respect to the non-ESBL-UPEC strains (44.32 *μ*g/ml). Of the fifty-two strains analyzed, only ten were resistant to fosfomycin (four strains of *Klebsiella pneumoniae* and six UPEC strains) at a maximum concentration of 160 *μ*g/ml; complete information is provided in Table 2.

When analyzing the urinalysis results, according to the uropathogen, we found significant differences for the presence of nitrites and bacteria (Table 3). We found that an acidic pH (5.2) was significantly different for UTIs due to *Klebsiella* after Bonferroni multiple comparison test (*p*=0.015). Cases due to *Proteus* seem to have a more basic pH. However, we only had a few cases, and it was not included in the statistical analysis. Almost all samples had leukocytes (94.7%) and leukocyte esterase (88.6%) irrespective of the uropathogen.

To identify clinical variables associated with the presence of ESBL- *E. coli*, we performed a multivariate analysis and found that the only clinical variable with significant association to the presence of ESBL producers was the presence of comorbidities, including hypertension and diabetes; both exhibited a significant association with an OR (IC95%) of 2.5(1.3 − 4.9)(*p* < 0.01) for hypertension and 2.8(1.2 − 6.7)(*p* < 0.05) for diabetes. None of the other clinical variables resulted in significant association to ESBL-*E. coli*, such as older age, feminine gender, pregnancy, menopause, previous UTIs, or hypothyroidism. After adjusting for confounding in a multiple variate model including all risk factors, the only variable that remain significant was hypertension OR (IC95%) of 2.5(1.1 − 5.7)(*p*=0.038).

## 4. Discussion

Main pathogens responsible for community-acquired uncomplicated UTIs were *E. coli* and ESBL-*E. coli*; however, the number of cases from the latter was lower compared to our own previously published findings (25% versus 68%) [11]. This discrepancy is highly due to differences in study design, since our previous published work was only based on laboratory reports database, and typically patients are only sent to the laboratory if they have recurrent infections or are not responding to treatment.

In the present work, we demonstrated an alarming resistance to commonly prescribed antibiotic drugs for uncomplicated UTIs (ampicillin, ceftriaxone, TMP-SMX, and ciprofloxacin).

When comparing our results to previous reports in other countries [22], we identified an even higher resistance to beta-lactams for ESBL–*E. coli* in our community. The presence of MDR genes including AmpC-*β*-lactamase, ESBLs, and carbapenemases is complicating the selection of empirical antimicrobial agents and is associated with treatment failure [23]. Previously published guidelines for UTIs recommend the initiation of treatment after a complete clinical evaluation and performance of urine analysis and cultures, for a judicious use of antibiotics, following local information on resistance patterns. There is also a general recommendation to avoid the use of antibiotics in asymptomatic bacteriuria; all of these measures will aid in a decreased exposition to antibiotics and avoid the rise in antimicrobial resistance rates [8, 24]. In our study, the most common clinical finding was pollakiuria (64%), and only 13% reported the presence of fever, which is consistent with a previous study, where the most frequent clinical finding was bladder tenderness in 93%, followed by pollakiuria in 92%, and the least common was fever in 7% of cases [7].

Some previously attributable risk factors for ESBL-producers infections are recurrence, older age, diabetes mellitus, female sex, urological procedures, prior use of antibiotics, anatomic anomalies, foreign material in the urinary tract, immunosuppression, and asymptomatic bacteriuria [23]. In our study, we confirmed diabetes in association with ESBL-*E. coli*, and even hypertension was associated with a higher strength. This association becomes remarkable, since we found two previous studies that reported *E. coli* bacteriuria with an increased risk for future hypertension development. Although the pathogenesis is not fully understood, there was a clear evidence of *E. coli*, a symptomatic bacteriuria implicated in the development of hypertension [25, 26]. Hypertension role should be further investigated in association with ESBL*-E. coli* colonization. Limitations of the present study include recall bias, since we did not have access to complete medical records; also we were not able to register response to treatment in previous UTIs.

### 4.1. Considerations for UTIs Treatment

We found a low proportion of resistance to fosfomycin even in the ESBL producers, probably due to its limited use in the American continent [12]. This bactericidal agent is a phosphoenolpyruvate analogue that binds covalently to the amino acid residue Cys155 of MurA and prevents the peptidoglycan biosynthesis, important for bacterial cell wall formation [27]. Fosfomycin is currently recommended as first-line treatment for uncomplicated UTIs due to the capability to interfere with the formation of biofilms; it can be used in combination with quinolones or aminoglycosides to increase their therapeutic effects [8]. Furthermore, fosfomycin exerts immunomodulatory effects on monocytes and T and B lymphocytes via production of inflammatory cytokines; nevertheless, results from different studies have been contradictory [28]. However, it was established that neutrophils incubated with fosfomycin were capable of increasing their bactericidal ability when challenged with ESBL-producing *E. coli* [29], although resistance has also been reported lately by genetic mutations in the chromosomally encoded transport systems in *Pseudomonas aeruginosa* [30], which explains our findings of fosfomycin resistance in cases due to *Pseudomonas*, *Klebsiella pneumoniae*, and *Proteus mirabilis*; although we had a limited number of cases, one previous study found intermediate resistance to fosfomycin in 13 isolates of enterococci (*n* = 6), *P. aeruginosa* (*n* = 5), and *Klebsiella* spp. (*n* = 2), and high resistance to fosfomycin in 5 isolates of *M. morganii* (*n* = 2), *P. aeruginosa* (*n* = 1), *Enterobacter aerogenes* (*n* = 1), and *Proteus mirabilis* [31].

On the other hand, nitrofurantoin remains a reliable agent for the empirical treatment of uncomplicated UTIs since most uropathogens were shown to be sensible. However, its use has been discouraged specially for the elderly due to its potential for pulmonary toxicity as reported by the American Geriatrics Society Updated Beers Criteria [32]. Additionally, nitrofurantoin does not achieve adequate serum or tissue levels and therefore should not be used in severe disease and is also contraindicated in pregnancy and patients with renal failure [8]. TMP-SMX and quinolones should be discarded as first options for the treatment of community-acquired UTIs due to *E. coli*, since the overall resistance rates are consistently high (above 20%) as recommended by the International Clinical Practice guidelines [12].

Furthermore, whenever there is suspicion of ESBL-*E. coli*, beta-lactams should be avoided, except for carbapenems and more recently developed beta-lactams such as ceftolozane-tazobactam and ceftazidime-avibactam [33]. In the case of *Enterococcus faecalis,* quinolones are a good therapeutic option in our media. Finally, carbapenems have the greatest level of activity against most uropathogens and have been recommended as first-line empiric therapy; nevertheless, we consider that their use should be restricted to serious infections, since its frequent use might be associated with colonization with carbapenem-resistant bacteria [34].

## 5. Conclusion

The most common pathogens being causative agents of community-acquired UTIs in our study were *E. coli* and ESBL-*E. coli*. For these, resistance rates to commonly prescribed antibiotic drugs were high, with the exception of fosfomycin, nitrofurantoin, and carbapenems. For other pathogens such as *Klebsiella* and *Proteus,* resistance rates to TMP-SMX and quinolones were low. Hypertension and diabetes were significantly associated with ESBL-*E. coli* in comparison to ESBL-non-producers. As a general recommendation, the identification of additional risk factors, such as the presence of diabetes or hypertension, could yield in a better selection of treatment, along with pathogen identification. Our results demonstrate that an active surveillance of resistance patterns in UTIs in the community is required since the proportion of ESBL producers represents a warning. Finally, the current use of fosfomycin in the American continent might be hampered due to lack of local in vitro susceptibility data and clinical experience; we expect that with the data provided in here, more clinical trials can be conducted to support and further explore the information presented.

## Figures and Tables

**Table 1:** Clinical and demographic variables of 296 patients with community-acquired urinary tract infection and asymptomatic bacteriuria.

Variable	Mean ± SD or *n* (%)
Age	54 ± 19
Feminine gender	262 (89)
Menopause	130 (44)
Pregnant	13 (4)
Multiparous (≥2 births)	128 (43)
Previous UTIs (≥1)	83 (28)

*Signs and symptoms*	*n* (%)
Pollakiuria	228 (77)
Change in urine color	187 (63)
Dysuria	181 (61)
Urinary urgency	170 (57)
Suprapubic pain	143 (48)
Hematuria	48 (16)
Fever	47 (16)
General malaise	22 (7)

*Comorbidities*	*n* (%)
Hypertension	66 (22)
Type 2 diabetes mellitus	25 (8)
Hypothyroidism	23 (8)

**Table 2:** Randomly selected UPEC strains to determine MIC and BIC to fosfomycin.

	*n*	*R*	*I*	*S*	MIC50 (*μ*g/mL)	MIC90 (*μ*g/mL)	MBC (*μ*g/mL)
		*n* (%)					
Non-ESBL UPEC	24	2 (8)	0 (0)	22 (92)	3.12	5.62	44.32
ESBL UPEC	24	4 (17)	0 (0)	20 (83)	3.21	5.78	63.75
*Klebsiella pneumoniae*	4	4 (100)	0 (0)	0 (0)	—	—	>160

*R*: resistant, *I*: intermediate, *S*: sensible, MIC: minimal inhibitory concentration, and MBC: minimal bactericidal concentration.

**Table 3:** Urinalysis findings in patients with UTIs according to the causative agent.

Urinalysis finding	*E. coli* (*n* = 164)	ESBL-*E. coli* (*n* = 76)	*Enterococcus* (*n* = 18)	*Klebsiella* (*n* = 14)	*p* value
pH mean ± SD	6.04 ± 0.7	5.8 ± 0.9	5.7 ± 0.8	5.2 ± 0.6	0.415
Positive nitrites *n* (%)	140 (85)	33 (43)	1 (6)	0 (0)	0.002
Positive proteins *n* (%)	46 (34)	12 (20)	5 (29)	2 (14)	0.154
Positive hemoglobin *n* (%)	26 (16)	9 (12)	2 (11)	3 (21)	0.798
LE (*μ*L) median (min-max)	75 (0–500)	100 (0–500)	50 (0–500)	50 (25–500)	0.745
WBCs (HPF) median (min-max)	75 (0–500)	38 (1–800)	30 (2–466)	27 (10–315)	0.993
RBCs (HPF) median (min-max)	1 (0–917)	12 (0–550)	2 (0–53)	0.5 (0–7)	0.558
Positive bacteria *n* (%)	62 (58)	30 (28)	4 (22)	11 (79)	0.048

## Data Availability

The data used to support the findings of this study are included within the article and within the supplementary information file(s).

## Supplementary Materials

Supplementary Materials Supplementary Table 1: main isolated pathogens (*n* ≥ 4) in urine samples from patients with community-acquired UTIs and their antimicrobial patterns.
