# Supplementary material for: The Clinical Significance of High Antimicrobial Resistance in Community-Acquired Urinary Tract Infections
Source: Can J Infect Dis Med Microbiol. 2020 Jun 4;2020:2967260. doi: 10.1155/2020/2967260 (PMC7293720; doi:10.1155/2020/2967260)
Supplement: Supplementary Materials — Supplementary Table 1: main isolated pathogens (n ≥ 4) in urine samples from patients with community-acquired UTIs and their antimicrobial patterns. [file 2967260.f1.docx]

**Supplementary Files**

Supplementary Table 1. Main isolated pathogens (n ≥ 4) in urine samples from patients with community acquired UTIs and their antimicrobial patterns.

| **Pathogen / Antibiotic** | | **Total** | | **Proportion n (%)** | | | |
| --- | --- | --- | --- | --- | --- | --- | --- |
|  |  | **n (%)** | | **S** | **I** | **R** | |
| ***E. coli n (%)*** | | **164 (55.4)** | | | | | |
| Beta-lactams | Ampicillin/Sulbactam | 163 | 73 (45) | | 0 (0) | | 90 (55) |
|  | Amoxicillin/Clavulanate | 10 | 5 (50) | | 3 (30) | | 2 (20) |
|  | Piperacillin/Tazobactam | 163 | 147 (90) | | 6 (4) | | 10 (6) |
|  | Cephalotin (1st) | 10 | 4 (40) | | 4 (40) | | 2 (2) |
|  | Cefuroxime (2nd) | 10 | 9 (90) | | 1 (10) | | 0 (0) |
|  | Ceftazidime (3rd) | 164 | 160 (98) | | 0 (0) | | 4 (2) |
|  | Ceftriaxone (3rd) | 164 | 159 (97) | | 0 (0) | | 5 (3) |
|  | Cefepime (4th) | 164 | 160 (98) | | 0 (0) | | 4 (2) |
|  | Imipenem | 154 | 154 (100) | | 0 (0) | | 0 (0) |
|  | Ertapenem | 154 | 154 (100) | | 0 (0) | | 0 (0) |
|  | Meropenem | 154 | 154 (100) | | 0 (0) | | 0 (0) |
| AG | Gentamicin | 164 | 140 (85) | | 0 (0) | | 24 (15) |
|  | Tobramycin | 164 | 138 (85) | | 17 (10) | | 9 (5) |
| QUIN | Ciprofloxacin | 163 | 111 (68) | | 1 (1) | | 51 (31) |
|  | Levofloxacin | 10 | 9 (90) | | 0 (0) | | 1 (10) |
|  | Moxifloxacin | 154 | 12 (62) | | 10 (6) | | 51 (32) |
|  | Nitrofurantoin | 162 | 151 (93) | | 8 (5) | | 3 (2) |
|  | Fosfomycin | 156 | 152 (98) | | 0 (0) | | 3 (2) |
|  | TMP/SMX | 162 | 90 (56) | | 0 (0) | | 72 (44) |
| ***ESBL- E. coli* n (%)** | | **76 (25.7)** | | | | | |
| Beta-lactams | Ampicillin/Sulbactam | 76 | | 8 (11) | 15 (20) | | 53 (69) |
|  | Piperaciclina/Tazobactam | 75 | | 59 (79) | 11 (15) | | 5 (6) |
|  | Ceftazidima | 76 | | 3 (4) | 0 (0) | | 73 (96) |
|  | Ceftriaxona | 76 | | 3 (4) | 0 (0) | | 73 (96) |
|  | Cefepime | 76 | | 4 (5) | 0 (0) | | 72 (95) |
|  | Imipenem | 76 | | 76 (100) | 0 (0) | | 0 (0) |
|  | Ertapenem | 75 | | 75 (100) | 0 (0) | | 0 (0) |
|  | Meropenem | 76 | | 76 (100) | 0 (0) | | 0 (0) |
| AG | Gentamicina | 76 | | 33 (43) | 0 (0) | | 43 (57) |
|  | Tobramicin | 76 | | 27 (36) | 14 (18) | | 35 (46) |
| QUIN | Ciprofloxacino | 75 | | 7 (9) | 1 (1) | | 67 (90) |
|  | Moxifloxacino | 76 | | 8 (11) | 0 (0) | | 68 (89) |
|  | Nitrofurantoin | 76 | | 72 (95) | 3 (4) | | 1 (1) |
|  | Fosfomicina | 76 | | 71 (93) | 0 (0) | | 5 (7) |
|  | TMP/SMX | 76 | | 35 (48) | 0 (0) | | 41 (54) |
| ***Enterococcus faecalis*** | | **18 (6.1)** | | | | | |
|  | Bencil-penicillin | 18 | | 15 (83) | 0 (0) | | 3 (17) |
|  | Ampicillin | 18 | | 15 (83) | 0 (0) | | 3 (17) |
|  | Streptomycin | 18 | | 13 (72) | 0 (0) | | 5 (28) |
|  | Gentamicin | 18 | | 14 (78) | 0 (0) | | 4 (22) |
| MC | Erythromycin | 18 | | 1 (5) | 10 (56) | | 7 (39) |
|  | Clindamycin | 18 | | 6 (33) | 0 (0) | | 12 (67) |
| QUIN | Ciprofloxacin | 18 | | 16 (89) | 0 (0) | | 2 (11) |
|  | Levofloxacin | 18 | | 16 (89) | 0 (0) | | 2 (11) |
|  | Moxifloxacin | 18 | | 10 (56) | 0 (0) | | 8 (44) |
|  | Quinupristine/Dalfopristine | 18 | | 1 (6) | 0 (0) | | 17 (94) |
|  | Nitrofurantoin | 17 | | 16 (94) | 1 (6) | | 0 (0) |
|  | Fosfomycin | 15 | | 15 (100) | 0 (0) | | 0 (0) |
|  | Linezolid | 15 | | 13 (87) | 0 (0) | | 2 (13) |
|  | Vancomycin | 18 | | 17(94) | 0 (0) | | 1 (6) |
| ***Klebsiella pneumoniae* n (%)** | | **14 (4.7)** | | | | | |
| Beta-Lactams | Ampicillin/Sulbactam | 13 | | 8 (62) | 0 (0) | | 5 (38) |
|  | Piperacillin/Tazobactam | 14 | | 11 (79) | 2 (14) | | 1 (7) |
|  | Ceftazidime | 13 | | 11 (85) | 0 (0) | | 2 (15) |
|  | Ceftriaxone | 14 | | 11 (79) | 0 (0) | | 3 (21) |
|  | Cefepime | 14 | | 11 (79) | 0 (0) | | 3 (21) |
|  | Imipenem | 13 | | 13 (100) | 0 (0) | | 0 (0) |
|  | Ertapenem | 13 | | 13 (100) | 0 (0) | | 0 (0) |
|  | Meropenem | 13 | | 13 (100) | 0 (0) | | 0 (0) |
| AG | Gentamicin | 14 | | 14 (100) | 0 (0) | | 0 (0) |
|  | Tobramycin | 14 | | 11 (79) | 0 (0) | | 3 (21) |
| QUIN | Ciprofloxacin | 14 | | 12 (86) | 2 (14) | | 0 (0) |
|  | Moxifloxacin | 13 | | 13 (93) | 0 (0) | | 0 (0) |
|  | Nitrofurantoin | 14 | | 7 (50) | 6 (43) | | 1 (7) |
|  | Fosfomycin | 12 | | 10 (83) | 0 (0) | | 2 (17) |
|  | TMP/SMX | 14 | | 11 (79) | 0 (0) | | 3 (21) |
| ***Staphylococcus aureus n (%)*** | | ***7 (2.4)*** | |  |  | |  |
|  | Gentamicin | 7 | | 7 (100) | 0 (0) | | 0 (0) |
| QUIN | Tigecycline | 7 | | 7 (100) | 0 (0) | | 0 (0) |
|  | Ciprofloxacin | 7 | | 4 (57) | 1 (14) | | 2 (29) |
|  | Moxifloxacin | 7 | | 6 (86) | 0 (0) | | 1 (14) |
|  | Nitrofurantoin | 7 | | 7 (100) | 0 (0) | | 0 (0) |
|  | Fosfomycin | 1 | | 1 (100) | 0 (0) | | 0 (0) |
|  | Clindamycin | 7 | | 2 (29) | 0 (0) | | 5 (71) |
|  | Vancomycin | 7 | | 7 (100) | 0 (0) | | 0 (0) |
| ***Pseudomonas aeruginosa n (%)*** | | ***6 (2.0)*** | |  |  | |  |
| Beta-Lactams | Ampicillin/Sulbactam | 5 | | 0 (0) | 0 (0) | | 5 (100) |
|  | Piperacillin/Tazobactam | 6 | | 4 (67) | 1 (17) | | 1 (17) |
|  | Ceftazidime | 6 | | 4 (67) | 0 (0) | | 2 (33) |
|  | Ceftriaxone | 6 | | 1 (17) | 1 (17) | | 4 (67) |
|  | Cefepime | 6 | | 4 (67) | 0 (0) | | 2 (33) |
|  | Imipenem | 5 | | 3 (60) | 0 (0) | | 2 (40) |
|  | Meropenem | 5 | | 3 (60) | 0 (0) | | 2 (40) |
| AG | Gentamicin | 6 | | 3 (50) | 0 (0) | | 3 (50) |
|  | Tobramycin | 6 | | 4 (67) | 0 (0) | | 2 (33) |
|  | Ciprofloxacin | 6 | | 3 (50) | 0 (0) | | 3 (50) |
|  | Moxifloxacin | 5 | | 2 (40) | 0 (0) | | 3 (60) |
|  | Nitrofurantoin | 6 | | 0 (0) | 1 (17) | | 5 (83) |
|  | Fosfomycin | 5 | | 2 (40) | 0 (0) | | 3 (60) |
|  | TMP/SMX | 6 | | 1 (17) | 0 (0) | | 5 (83) |
| ***Proteus mirabilis n (%)*** | | ***4 (1.4)*** | |  |  | |  |
| Beta-Lactams | Ampicillin/Sulbactam | 2 | | 2 (100) | 0 (0) | | 0 (0) |
|  | Piperacillin/Tazobactam | 4 | | 4 (100) | 0 (0) | | 0 (0) |
|  | Ceftazidime | 4 | | 4 (100) | 0 (0) | | 0 (0) |
|  | Ceftriaxone | 4 | | 4 (100) | 0 (0) | | 0 (0) |
|  | Cefepime | 4 | | 4 (100) | 0 (0) | | 0 (0) |
|  | Ertapenem | 2 | | 2 (100) | 0 (0) | | 0 (0) |
|  | Meropenem | 2 | | 2 (100) | 0 (0) | | 0 (0) |
| AG | Gentamicin | 4 | | 3 (75) | 0 (0) | | 1 (25) |
|  | Tobramycin | 4 | | 3 (75) | 0 (0) | | 1 (25) |
|  | Ciprofloxacin | 4 | | 3 (75) | 1 (25) | | 0 (0) |
|  | Moxifloxacin | 2 | | 1 (50) | 0 (0) | | 1 (50) |
|  | Nitrofurantoin | 4 | | 0 (0) | 0 (0) | | 4 (100) |
|  | Fosfomycin | 3 | | 2 (67) | 0 (0) | | 1 (33) |
|  | TMP/SMX | 4 | | 3 (75) | 0 (0) | | 1 (25) |

AG Aminoglycosides, QUIN quinolones, MC macrolides, TMP/SMX trimethoprim sulfamethoxazole
